# Novel insights on carbon source influence on azomycin production by *Streptomyces eurocidicus*

**DOI:** 10.1007/s42770-026-02102-8

**Published:** 2026-10-12

**Authors:** Julia de Macedo Robert, Wagner Lopes, Alexandre Andrade de Souza Costa, Otavio Padula de Miranda, Tatiane Aparecida Nascimento, Maria da Conceição Avelino Dias, Filipe Soares Quirino da Silva, Fabius Vieira Leineweber, Graziela Jardim Pacheco

**Affiliations:** 1 Bioprocess Laboratory, Institute of Drug Technology, Oswaldo Cruz Foundation, Fiocruz, Rio de Janeiro, Brazil; 2 Integrated Laboratory of Simuliids and Onchocerciasis & Medical and Forensic, Oswaldo Cruz Institute, Oswaldo Cruz Foundation, Fiocruz, Rio de Janeiro, Brazil; 3 Drug Synthesis Laboratory – Pharmacokinetics Unit, Institute of Drug Technology, Oswaldo Cruz Foundation, Fiocruz, Rio de Janeiro, Brazil

**Keywords:** Azomycin, Streptomyces, Mixed substrates, Fermentation, Antibiotic, Natural products

## Abstract

Azomycin (2-nitroimidazole), a compound synthesized by *Streptomyces eurocidicus*, is medically significant as a build block for the drug applied in treatment for drug-resistant tuberculosis and Chagas disease, as well as tumor hypoxia imaging. Despite its clinical value, the optimized bioprocess parameters for its production remain largely unexplored. This study evaluated the influence of various carbon and nitrogen sources (specifically glucose, starch, soybean oil, and casamino acids) on azomycin yields during submerged cultivation. Batch fermentations revealed that azomycin biosynthesis is phase-shifted, initiating only after the primary carbon source is near depletion. Notably, although soybean oil served as an excellent carbon source for biomass accumulation, it yielded no detectable azomycin production. By contrast, supplementing the media with free amino acids (casamino acids) substantially enhanced product synthesis. Using an optimized semi-defined medium of glucose and casamino acids, a titer of 46.34 mg/L was achieved in 250 mL DASbox system. Scaling this configuration to an instrumented 3 L bioreactor resulted in a final concentration of 69.18 mg/L over 7 days, marking an 89% increase in the product-to-biomass yield (Yp/x) compared to the smaller scale tested. Liquid chromatography-mass spectrometry (LC-MS) verified the identity of the extracted 2-nitroimidazole. These findings demonstrate that the precision balancing of glucose and organic nitrogen is essential to unlock and maximize the biosynthetic potential of wild-type S. eurocidicus.

**Supplementary Information:**

The online version contains supplementary material available at https://doi.org/10.1007/s42770-026-02102-8.

## Introduction

The genus *Streptomyces* comprises a diverse group of Gram-positive, filamentous actinomycetes widely recognized as one of the most prolific producers of bioactive secondary metabolites [1]. These soil-dwelling microorganisms exhibit a complex developmental cycle characterized by the formation of substrate mycelium, the emergence of aerial hyphae, and the production of spore chains [2]. Ecologically, *Streptomyces* species play an essential role in nutrient cycling and microbial competition through the secretion of extracellular enzymes and specialized metabolites [1]. From an industrial perspective, they remain the primary source of clinically relevant antibiotics, accounting for approximately two-thirds of all known antibiotics in use, including streptomycin, tetracycline, erythromycin, and chloramphenicol [3]. In addition to antibiotics, *Streptomyces* species synthesize a wide range of secondary metabolites with diverse biological activities, such as immunosuppressants, antifungals, anticancer agents, and agricultural biocontrol compounds [3, 4]. This remarkable metabolic diversity is encoded by numerous biosynthetic gene clusters (BGCs), many of which remain cryptic under standard laboratory conditions, suggesting that the full biosynthetic potential of this genus is far from fully explored [4].

Among *Streptomyces* species, *Streptomyces eurocidicus* attracted attention in the early 1960’s due to its ability to produce structurally diverse secondary metabolites with significant biological activities, including tertiomycin, azomycin, and eurocidin [5]. Despite these initial discoveries, contemporary data on its physiology, metabolic regulation, and performance under controlled cultivation conditions remain scarce. This lack of modern bioprocess characterization limits a deeper understanding of its metabolic capabilities and constrains efforts to design and optimize efficient production processes.

Azomycin (2-nitroimidazole) stands out as a significant metabolite produced by *S. eurocidicus* due to its considerable pharmaceutical relevance. Compounds within the 2-nitroimidazole class are employed in the treatment of drug-resistant tuberculosis [6], and their derivatives have found applications in both therapy and diagnostics. One notable example is benznidazole, developed in 1972, which remains one of the few effective treatments for *Trypanosoma cruzi*, the causative parasite of Chagas disease [7]. Additionally, 2-nitroimidazole derivatives are widely used as imaging agents for tumor hypoxia, an important indicator of chemotherapy resistance [8–10] and have demonstrated promising antitumor activity under hypoxic conditions [11, 12]. These applications underscore the need for reliable and scalable strategies for azomycin production.

Investigations into the azomycin biosynthetic pathway identified 2-aminoimidazole as a key intermediate. This compound was observed to accumulate in the culture medium and, to a lesser extent, in the mycelium, supporting its role as a precursor of 2-nitroimidazole based on its in vivo conversion [13]. Two other biochemical evidence demonstrated that L-arginine serves as the primary precursor, being converted to azomycin via 2-aminoimidazole in the producing strain [14, 15]. These studies identified a cryptic azomycin biosynthetic gene cluster in *Streptomyces cattleya.* The pathway involves the sequential action of RohP, RohR, RohQ, and RohS enzymes, leading L-arginine to the formation of the azomycin [15].

Despite the early discovery of azomycin and partial elucidation of its biosynthetic pathway [13, 14], and recent confirmation of the complete route [15], studies addressing its production under controlled bioprocess conditions remain limited. A Table with the reported azomycin production data is presented in Supplementary materials (Table S1). While historical reports established *S. eurocidicus* as an azomycin producer, quantitative information regarding cultivation performance, substrate utilization, and azomycin yields under controlled bioprocess conditions remains scarce. In particular, the influence of cultivation parameters on metabolite yield has not been thoroughly explored. Achieving maximum metabolite yields in wild-type precision fermentation requires understanding of how media composition, particularly availability of carbon and nitrogen sources, influences the regulatory mechanisms governing antibiotic biosynthesis [16].

In this context, the present study investigates azomycin (2-nitroimidazole) production by *S. eurocidicus* under submerged cultivation, with particular focus on evaluating the effect of different carbon sources on process performance.

## Materials and methods

### Materials

All reagents used in this study were of analytical grade or higher. Glucose (Sigma-Aldrich, USA), yeast extract (Oxoid, UK), malt extract (Kasvi, Brazil), calcium carbonate (ACS, Brazil), agar (Dinâmica, Brazil), sodium phosphate monobasic (NaH₂PO₄) (Sigma-Aldrich, Japan), dipotassium phosphate (K₂HPO₄) (Cinética, Brazil), ammonium sulfate (NH₄)₂SO₄ (Sigma-Aldrich, USA), soluble starch (Êxodo cientifica, Brazil), soybean oil (Bunge, Brazil), casamino acids (Sigma-Aldrich, USA), zinc sulfate heptahydrate (ZnSO₄·7 H₂O) (Sigma-Aldrich, India), iron sulfate heptahydrate (FeSO₄·7 H₂O) (Sigma-Aldrich, India), manganese chloride tetrahydrate (MnCl₂·4 H₂O) (Sigma-Aldrich, USA), calcium chloride dihydrate (CaCl₂·2 H₂O) (Merck, Germany), sodium hydroxide (NaOH) (Merck, Germany), sulfuric acid (H₂SO₄) (Merck, Germany), Triton X-100 (Sigma-Aldrich, USA), ethyl acetate (Synth, Brazil), dimethyl sulfoxide (DMSO) (Êxodo, Brazil), methanol (Merck, Germany), acetonitrile (Merck, Germany), formic acid (Merck, Germany).

As analytical reference standard for HPLC quantification an *in-house* purified sample with 95% purity 2-nitroimidazole was used (confirmed by LC-MS).

For electron microscopy sample preparation, glutaraldehyde (Electron Microscopy Sciences, USA), sodium cacodylate (Electron Microscopy Sciences, USA), poly-L-lysine (Sigma-Aldrich, United Kingdom), osmium tetroxide (Electron Microscopy Sciences, USA), ethanol (Merck, Germany), Epon resin (Electron Microscopy Sciences, USA), uranyl acetate (Electron Microscopy Sciences, USA), lead citrate (Electron Microscopy Sciences, USA), aluminum stubs (Koch Electron Microscopy Ltda., Brazil), double-sided adhesive conductive carbon tape (Ted Pella Inc., Redding, CA, USA), and gold coating target (Ted Pella Inc., Redding, CA, USA) were used.

### Microorganism and stock culture

This study employed *Streptomyces eurocidicus* DSM 40,604, obtained from the Leibniz Institute DSMZ–German Collection of Microorganisms and Cell Cultures GmbH. The strain was reactivated according to the supplier’s instructions, and the master spore bank was established and stored in an aqueous solution containing 50% (v/v) glycerol at − 80 °C.

For routine experimental use, a working spore stock was prepared from spores harvested from *S. eurocidicus* grown on GYM agar medium (glucose, 4 g L⁻¹; yeast extract, 4 g L⁻¹; malt extract, 10 g L⁻¹; calcium carbonate, 2 g L⁻¹; agar, 20 g L⁻¹) for 15 days. Spores were gently scraped from Petri dishes using a sterile inoculation loop and suspended in sterile purified water. The resulting spore suspension was aliquoted into 1 mL vials and stored at 4 °C until use.

### Instrumented mini-bioreactor cultivation

For experiment initiation, spores stored under refrigerated conditions were cultivated in an orbital shaker (Innova 44, New Brunswick Scientific, USA) at 28 °C and 150 rpm for 3 days in the same culture medium used in each mini-bioreactor (DASbox, Eppendorf, Germany), with a working volume of 250 mL. Culture media were prepared as follows:

- The basal medium (per liter) contained 1.8 g NaH_2_PO₄, 2.6 g K₂HPO₄, 3.0 g (NH₄)₂SO₄, 10 g of the carbon source, and 1 mL of trace salt solution.
- The trace salt solution (per liter) contained 1.0 g ZnSO₄·7 H₂O, 1.0 g FeSO₄·7 H₂O, 1.0 g MnCl₂·4 H₂O, and 1.0 g CaCl₂·2 H₂O.

The carbon source varied across experiments and consisted of glucose, soluble starch, soybean oil, casamino acids, or their combinations. When more than one carbon source was used, the total carbon source concentration was maintained at 10 g L⁻¹.

The trace salt solution was sterilized by filtration through a 0.22 μm PES membrane (KASVI, China) and stored at 4 °C protected from light until use. Culture media were sterilized by autoclaving at 121 °C for 30 min. When required for foam control, antifoam Sigma 204 (Sigma-Aldrich, USA) was applied as a 10-fold diluted solution in distilled water and sterilized under the same autoclaving conditions prior to use.

The mini-bioreactors were inoculated with 20 mL of the shaker-grown culture. Cultivations were carried out at 28 °C with agitation and aeration rates ranging from 150 to 400 rpm and 0 to 1 vvm, respectively, under cascade control to maintain the dissolved oxygen at approximately 30%. Each DASbox system vessel is equipped with two 6-blades Rushton impellers and distance between the two is 23 mm. The pH was controlled at 7.0 using 2 M NaOH. An initial sample was collected at the start of the fermentation and another one at the end, after 7 days of growth.

### Bioreactor cultivation

A BioFlo 310 bioreactor (New Brunswick Scientific) was operated at a working volume of 3 L. The selected media used was a semi-defined media containing, per liter, 2.5 g Glucose, 5 g casamino acids, 1.8 g NaH_2_PO₄, 2.6 g K₂HPO₄, 3.0 g (NH₄)₂SO₄, and 1 mL of trace salt solution. Trace salt solution had the same composition as previously described. The bioreactor ran at 28 °C, with stirrer and aeration controlled in cascade mode (DO 30%), ranging from 150 to 400 rpm and 0–1 vvm, respectively. The vessel was equipped with two 6-blades Rushton impellers and distance between the two is 60 mm. The pH was maintained at 7.0 using 2 M NaOH. Inoculation involved adding 300mL of pre-inoculum grown in shaker at 28 °C 150 rpm for 2 days using the same media. Samples were collected each day over a 7-day period.

### Dry cell weight analysis

The final sample of the process was subjected to dry cell weight analysis. A 0.22 μm cellulose acetate membrane (Sartorius Stedim Biotec, Germany) was pre-weighed and subsequently used to vacuum-filter 10 mL of the culture sample. The membrane containing the retained cells was dried in an oven at 70 °C for 16 h or until constant weight was achieved. Prior to weighing, the membrane was allowed to equilibrate to room temperature with all measurements performed in triplicate.

### Triglyceride analysis

Triglyceride concentration in the culture medium was determined using a commercial enzymatic kit BIOCLIN K117-2 (BIOCLIN, Brazil), following the manufacturer’s instructions [17]. Prior to analysis, each sample received 0.1% Triton X-100 to improve homogenization of the lipid fraction. Subsequently, a 2.5 µL aliquot of the reaction mixture was combined with 250 µL of the enzymatic reagent in a well of a 96-well microplate and incubated at 37 °C for 10 min. Absorbance was measured at 500 nm. All samples were analyzed in triplicate.

### Quantitative glucose determination

Glucose concentration in the culture medium was measured using a OneTouch Select Plus Flex glucometer (LifeScan, USA), following the manufacturer’s instructions. Each sample was analyzed in triplicate.

### Quantitative starch determination

Starch concentration was determined by measuring the total glucose released after acid hydrolysis of the samples, following an adapted protocol previously described in the literature [18, 19]. Briefly, 2 mL of the sample were transferred to a screw-cap glass tube and mixed with 2 mL of 1.5 M H₂SO₄. The hydrolysis reaction was carried out at 80 °C for 2 h.

After completion of the reaction, 2 mL of 5 M NaOH were added to neutralize the solution. For glucose quantification a commercial enzymatic kit BIOCLIN K082 (BIOCLIN, Brazil) was used. A 2.5 µL aliquot of the hydrolyzed sample was mixed with 250 µL of an enzymatic reagent in a well of a 96-well microplate, in triplicate. The plate was incubated at 37 °C for 10 min, and the absorbance was measured at 505 nm.

### Quantitative organic nitrogen determination

Determination of free alpha-amino terminal groups in fermentation was performed according to the methodology described by the European Brewery Convention, 1987 [20] which specifies the composition of the solutions used. A sample was diluted so that the final absorbance of the colorimetric reaction fell within the standard curve range. In each test tube, 1 mL of sample was added, followed by 500 µL of the reagent solution, and then placed in a water bath at 100 °C for 16 min. After cooling, 5 mL of dilution solution was added to the tubes. The blank was prepared using 1 mL of distilled water instead of the sample. Absorbance was measured in triplicate using a microplate reader at 570 nm. The conversion factor obtained from the standard curve was 0.0791 mg/L.

### Determination of the initial carbon-to-nitrogen (C/N) ratio

The initial carbon-nitrogen (C/N) mass ratio of media used were calculated based on the elemental carbon and nitrogen content of each variation evaluated. Carbon and nitrogen mass fractions of pure compounds (glucose, ammonium sulfate and soluble starch) were calculated from their molecular formula, according to Eq. 1:

1 $$\:\%C\:or\:\%N=\frac{n\ast M_{at}}{{PM}_{mol}}\ast100$$

Where n is the number of carbon or nitrogen per molecule, M_at_ is the atomic mass of the element and PM_mol_ is the molecular weight of each molecule.

For soybean oil it was considered 78% of carbon content in the total weight and no nitrogen present. For casaminoacids, the elemental nitrogen content was considered the value measured by the quantitative organic nitrogen described above and, for carbon content, 40% was used as this information was not available on the certificate of analysis for this specific product. Total carbon and nitrogen concentrations in the medium were obtained by summing the individual contributions from all carbon and nitrogen containing components and the ratio calculated according to Eq. 2:

2 $$\:C/N\:(g/g)=\:\frac{\sum\:C_i\ast\%C}{\sum\:C_i\ast\%N}$$

Where C_i_ is the initial concentration of the component in the media (g/L).

### Calculation of specific power input (P/V)

The volumetric power input (P/V) delivered by the impeller over the course of both cultivations (3 L and 250 mL bioreactor) was calculated point-by-point since both stirring speed and gasflow were adjusted by a cascade control loop in response to dissolved oxygen demand. For each control interval, the ungassed power number (P_0_) was first calculated using the standard turbulent-regime expression for a Rushton turbine with baffles:

3 $$\:{P}_{0}={Np}_{0}*\rho\:*{N}^{3}*{Di}^{5}*n$$

Where Np_0_ is the power number of the impeller (5), ρ is broth density (kg/m^3^), N is the stirring speed (rps), Di is impeller diameter (m), and n is the number of impellers on the vessel. Flow regime was confirmed as turbulent for all conditions.

The gassed power (P_g_) was estimated from P_0_ using empirical correlation of Michel and Miller [21]:

4 $$\:{P}_{g}=C*{\left(\frac{{P}_{0}^{2}*N*{Di}^{3}}{{Q}^{\mathrm{0,56}}}\right)}^{0.45}$$

Where Q is the volumetric gasflow rate (m^3^/s) and C is an empirical constant accounting for impeller/vessel geometry (1). Under non-aerated conditions, P_g_ was taken as equal to P_0_.

Specific power input was then calculated where V was the working culture volume (m^3^) and expressed in W/m^3^. Because N and Q varied continuously through cultivation according to the cascade set points, P/V was computed for each corresponding N-Q pair recorded by the bioreactor control system, yielding a time-resolved P/V profile rather than a single fixed value. An unaccounted source of uncertainty for the 250 ml scale is that, as the vessel was operated without baffles, so the values reported for this scale should be regarded as upper estimates. All calculations were performed at Microsoft Excel.

### Ethyl acetate extraction and HPLC analysis of 2-nitroimidazole

Approximately 40 mL of the aqueous culture medium extract containing 2-nitroimidazole (2-NI) produced by *S. eurocidicus* was subjected to liquid–liquid organic partitioning in a separatory funnel at 25 °C and pH 2.0. Ethyl acetate served as the organic solvent due to its low water miscibility and high affinity for 2-nitroimidazole. After extraction, the organic phases were pooled and evaporated to dryness under reduced pressure at temperatures ranging from 60 to 80 °C.

The resulting solid residue was dissolved in 0.5 mL of dimethyl sulfoxide (DMSO) and 4.5 mL of methanol. The solution was brought to a final volume of 5.0 mL, filtered through a 0.22 μm membrane filter, and a 5 µL aliquot was injected into the HPLC system for determination of the 2-nitroimidazole concentration. Quantification was performed by comparison with a reference solution prepared from 95% pure 2-NI at a known concentration, using the same 5 µL injection volume. Under the chromatographic conditions employed, 2-NI was identified based on a retention time window of 7.3–7.5 min. Raw data of the analytical standard (Fig. S1) and one example of fermentation sample (Fig. S2) is available at Supplementary materials.

The analytical method followed an adapted procedure described in the literature [22]. Chromatographic analyses were performed using a VWR-Hitachi Chromaster HPLC system comprising five modules: a quaternary pump (5160), autosampler (5260), column oven (5310), diode array detector (DAD, 5430), and organizer. Detection of 2-NI was carried out at a wavelength of 324 nm using a YMC-Triart C18 column (250 × 4.6 mm, 5 μm particle size, 120 Å pore size), for which a retention factor (k′) of 1.3 was observed.

The mobile phases consisted of Phase A: 0.1% (v/v) aqueous formic acid solution, and Phase B: acetonitrile containing 0.1% (v/v) formic acid. The chromatographic separation was conducted under a binary gradient elution program as follows: (95%A: 5%B) 0,0→5,0 min; (0%A; 100%B) 5,0→6,0 min; (0%A; 100%B) 6,0→14,0 min; (95%A; 5%B) 14,0 →15,0 min, (95%A; 5%B) 15,0 →20,0 min. Analyses were performed at 25 °C with a flow rate of 1.0 mL min⁻¹. The method exhibited a limit of quantification (LOQ) of 0.1 mg L⁻¹.

### Identification of 2-nitroimidazole in fermented broth

Liquid chromatography–mass spectrometry (LC–MS) was employed to identify 2-nitroimidazole in the fermented broth. Samples were prepared as described in Sect.  2.2, with methanol replaced by acetonitrile in the final step. Samples were analyzed in a LCMS 9050 system, from Shimadzu. An Acquity HSS T3 column (2,1 × 100 mm; 1,8 μm), from Waters was used. Mobile phases were water with 0,1% formic acid (A) and acetonitrile with 0,1% formic acid (B), flow rate 0,3mL/min. Gradient was (97%A: 3%B) 0,00→1,00 min ; (97%A: 3%B) 1,00→1,01 min; (10%A: 90%B) 1,01→9,00 min; (10%A: 90%B) 9,00→ 10,00 min; (97%A: 3%B) 10,00→10,01 min; (97%A: 3%B) 10,01→12,00 min. An electron spray ionization source (ESI) was used in the mass spectrometer, and the equipment was programed to acquire data in both positive and negative modes. All data was acquired using Labsolution and processed with Labsolution Insight, both from Shimadzu.

### Scanning electron microscopy (SEM)

Scanning electron microscopy (SEM) analysis examined the morphology and dimensions of the crystals detected in *S. eurocidicus* strains. The methodology followed previously described protocols [23]. Culture supernatants and pellets were obtained from fermentation containing glucose and soybean oil as carbon sources on semi-defined media. The presence of crystal complexes was initially confirmed using a phase-contrast microscope (Zeiss Axioskop, Germany) at 1000× magnification.

After incubation, the bacterial cultures were centrifuged at 14,000 rpm for 5 min. The resulting bacterial pellets were fixed in 2.5% glutaraldehyde prepared in 0.01 M sodium cacodylate buffer for 1 h at room temperature.

Subsequently, the material was allowed to adhere to poly-L-lysine–coated coverslips for 48 h in tissue culture plates. The samples were then washed three times with 0.01 M sodium cacodylate buffer and post-fixed with 0.5% osmium tetroxide for 1 h. After post-fixation, the samples were dehydrated through a graded ethanol series (25, 50, 75, 90, and 100%), with two immersions at each concentration for 20 min.

The dehydrated samples were dried using the critical point drying method at the Rudolf Barth Electron Microscopy Platform, Oswaldo Cruz Institute (FIOCRUZ). Following this step, the coverslips were mounted onto aluminum stubs using double-sided adhesive tape and returned to the microscopy platform for metal coating. The specimens were sputter-coated with gold and examined using a JEOL JSM-6390LV scanning electron microscope at the Rudolf Barth Electron Microscopy Platform, Oswaldo Cruz Institute. The micrographs were recorded and stored on CD-RW media for subsequent analysis.

### Transmission electron microscopy (TEM)

The bacterial pellet was fixed and post-fixed according to the standard protocol previously described for bacterial sample preparation for scanning electron microscopy (SEM). Culture pellets were obtained from fermentation containing soybean oil as carbon source.

Subsequently, the material was infiltrated with a mixture of absolute ethanol and epoxy resin (Epon) at a ratio of 2:1 for 4 h, followed by a 1:1 ratio for 16 h. The samples were then embedded in pure Epon for 24 h and polymerized in an oven at 60 °C for 72 h. The resulting blocks were processed at the Rudolf Barth Electron Microscopy Platform, Oswaldo Cruz Institute, where ultrathin sections were obtained using a Leica Ultracut S ultramicrotome. The sections were contrasted with 5% uranyl acetate in complete darkness and subsequently with 2% lead citrate.

The ultrathin sections were examined using a JEOL JEM-1011 transmission electron microscope at the Rudolf Barth Electron Microscopy Platform, Oswaldo Cruz Institute. The micrographs were recorded and stored on CD-RW media for subsequent analysis.

## Results

**Fig. 1:**
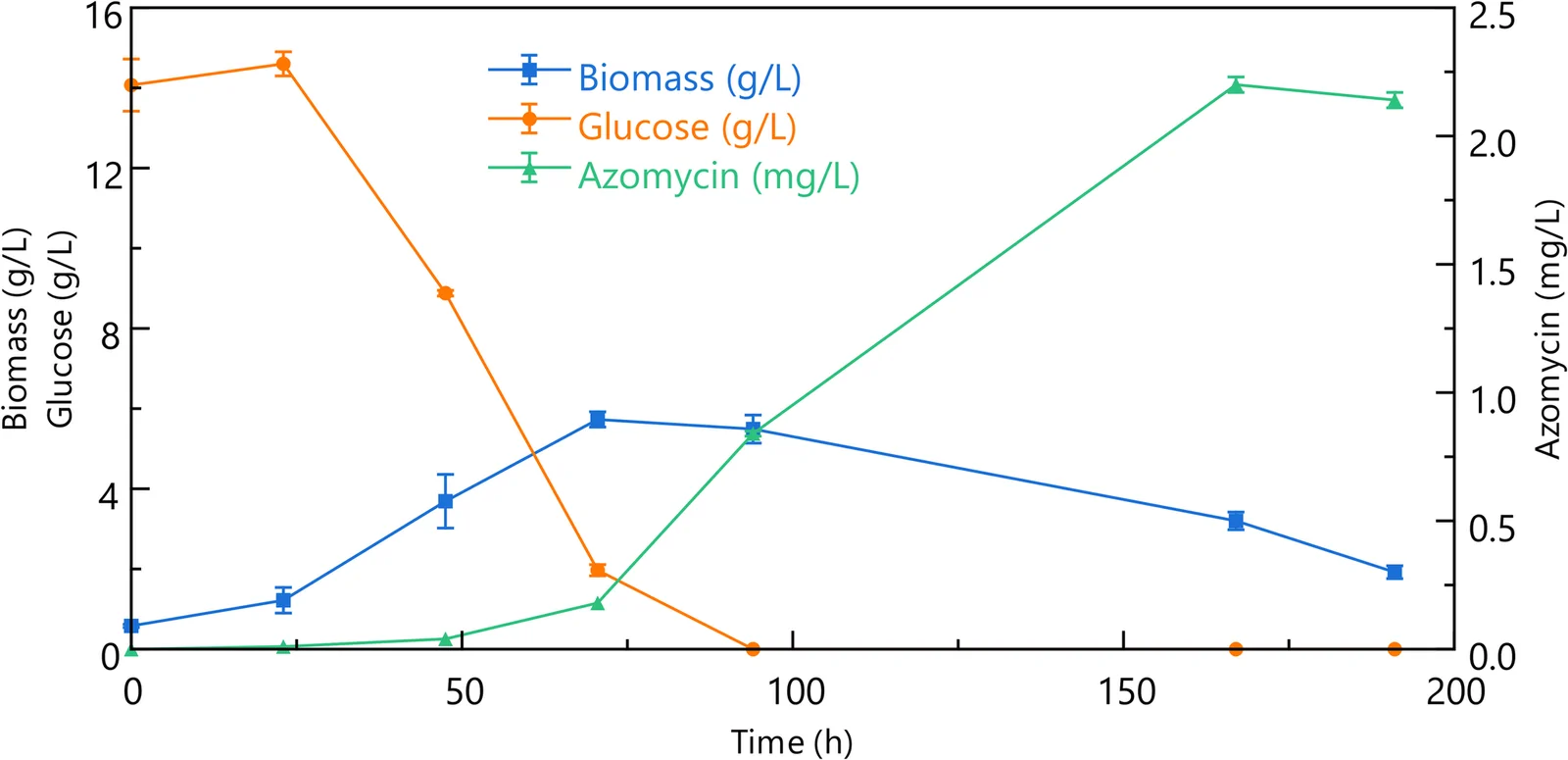
Biomass growth, glucose consumption and azomycin production through 192 h cultivation of *S. eurocidicus* on 3 L bioreactor using minimal media with glucose as carbon source. Data from a single run, error bars calculated by triplicate analysis. Concentration shown as: ■ Biomass (g/L); ● Glucose (g/L); ▲Azomycin (mg/L)

First, an exploratory culture was conducted in the 3 L bioreactor to provide a comparison basis for production behavior and to establish the most suitable time points, as this information was not available in previous literature for this specific species. The results are shown on Fig. 1.

In this experiment, azomycin production starts between 48 and 72 h of culture which coincides with glucose being almost depleted at 72 h and biomass growth slowdown from exponential phase, normally where the metabolism shift occurs while the primary metabolism is still active.

**Fig. 2:**
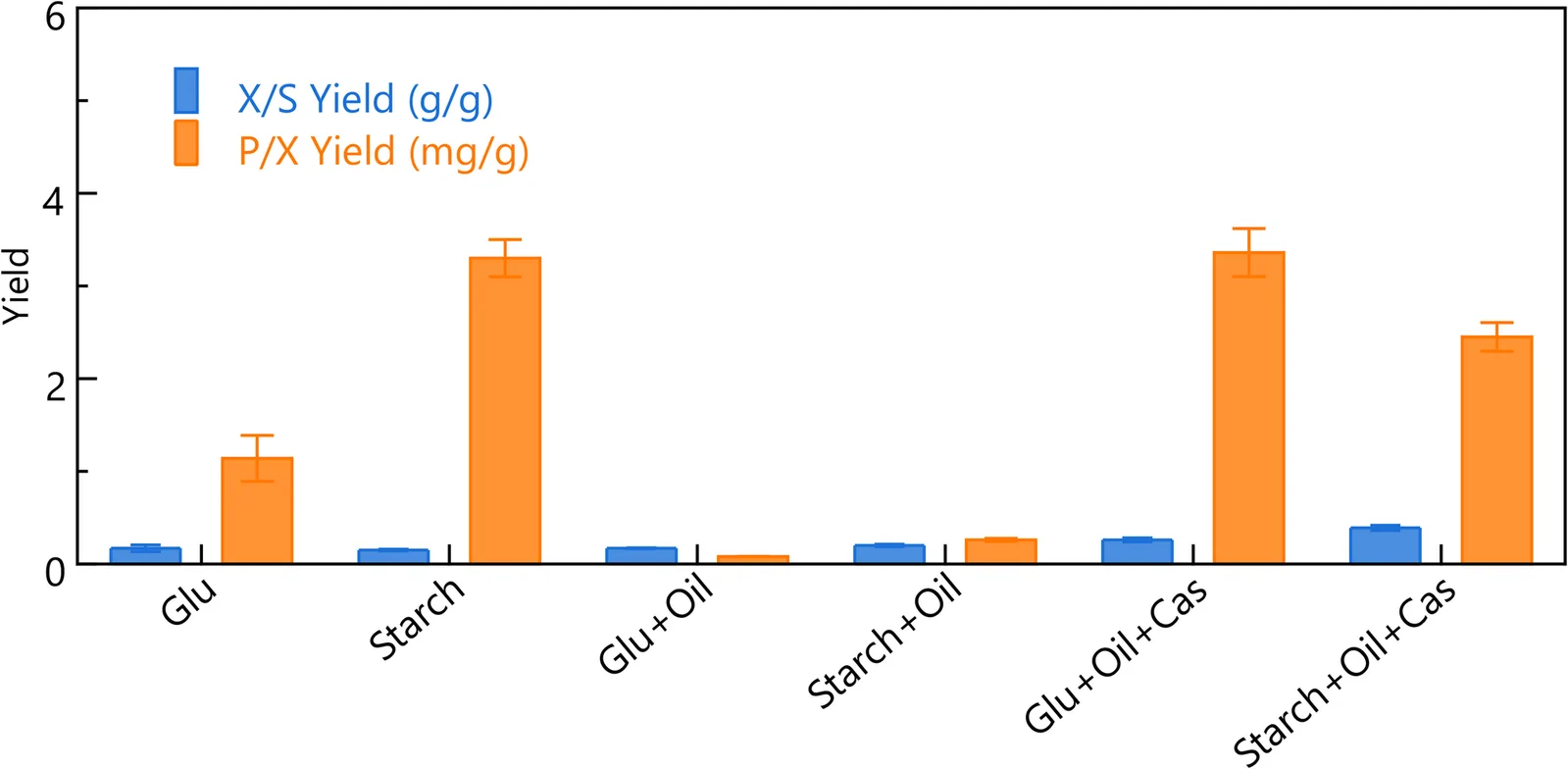
Azomycin per biomass (orange) and biomass per substrate (blue) yields obtained after 168 h of growth on minibiorector using different carbon sources: Glucose (10 g/L); Starch (10 g/L); Glucose (5 g/ L) and soybean oil (5 g/L); Starch (5 g/L) and soybean oil (5 g/L); Soybean oil (5 g/L) and casamino acids (5 g/L); Glucose (2.5 g/L), soybean oil (2.5 g/L) and casamino acids (5 g/L); Starch 2.5 (g/L), soybean oil (2.5 g/L) and casamino acids (5 g/L), respectively. Exact numerical values are provided in Table S2 for comparison

After this base result, some media modifications were made in order to explore its behavior. First, the use of starch and soybean oil as sole carbon source, then a mix between them, followed by the addition of casamino acids that aimed to enhance the azomycin production. In the last approach, the total carbon source concentration was split and half of it was substituted by casamino acids (5 of the 10 g/L). Based on these modifications, growth, consumption and azomycin production were tested in a minimum media, followed by the mix on a semi-defined media containing casamino acids. The yields obtained in each bioprocess are shown in Fig. 2.

In addition to the variations presented in Fig. 2, an oil-only experiment was conducted, but substrate consumption could not be accurately determined due to sampling difficulties associated with the fully emulsified oil at the stirring speed used. Nevertheless, the culture reached a twofold higher biomass concentration. While other conditions ranged between 1.7 and 2.0 g/L at 168 h, this condition reached 3.5 g/L. No azomycin production was detected and therefore these results were not included in the image. As for the remaining condition, Yx/s ranged from 0.15 to 0.2 g/g in the absence of casamino acids, increasing to 0.3–0.4 g/g when present. Regarding the azomycin production-per-biomass yield among single carbon sources, starch yielded a 2.8-fold higher value than glucose alone. However, this pattern did not persist after adding soybean oil and casamino acids. In the mixed substrate conditions, media containing glucose resulted in a higher Yp/x (1.4-fold higher) than media containing starch.

**Fig. 3:**
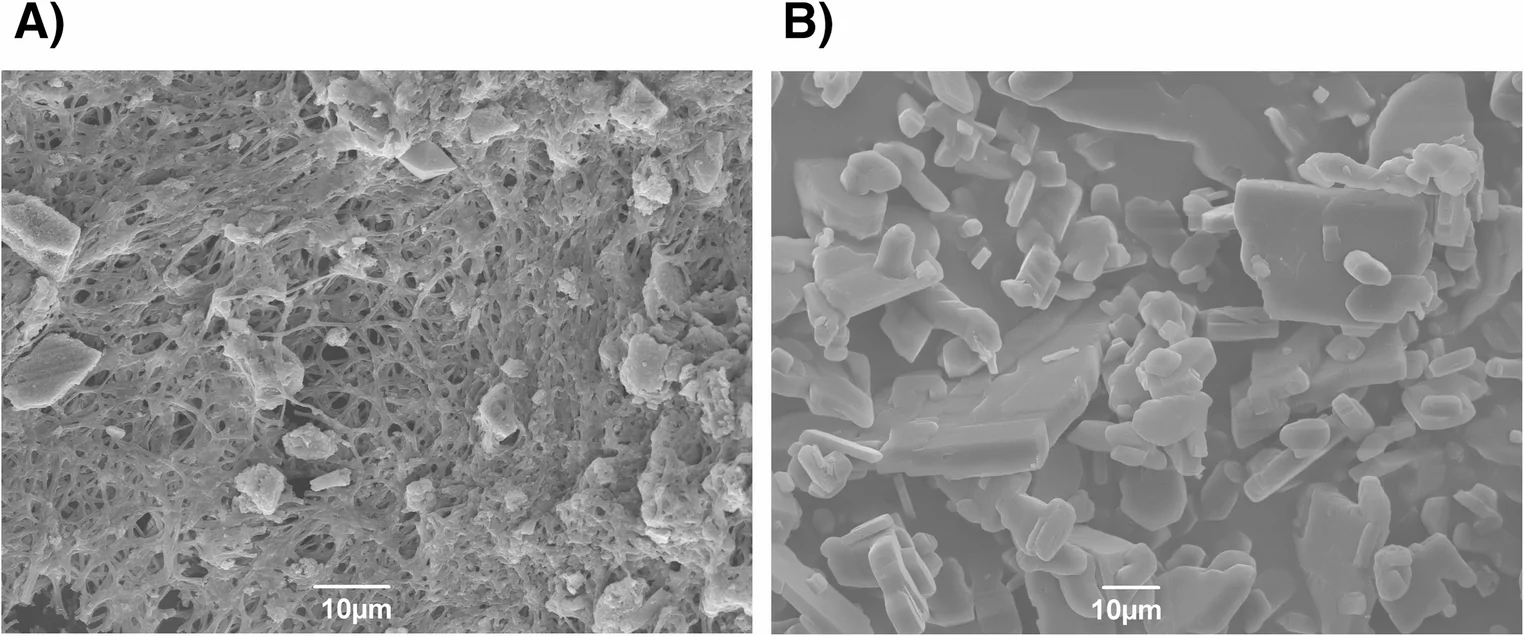
Scanning Electron Microscopy (SEM) images of (**A**) sample of *S.eurocidicus* after 168 h growth on glucose+soybean oil+casamino acids; and (**B**) sample of standard azomycin

On the microscopies made to ensure purity of the culture (data not shown) was observed that the presence of casamino acid led to the presence appearance of crystal structures as shown in Fig. 3.

In Fig. 3a, crystals within the pellet structure can be observed in various sizes, predominantly exhibiting rectangular cuboid shape. These structures also appear dispersed in the supernatant and were not present in the other media tested in this initial screening (data not shown). A comparison with the SEM image of a standard azomycin solution (Fig. 3b) shows that crystals are morphologically similar.

**Fig. 4:**
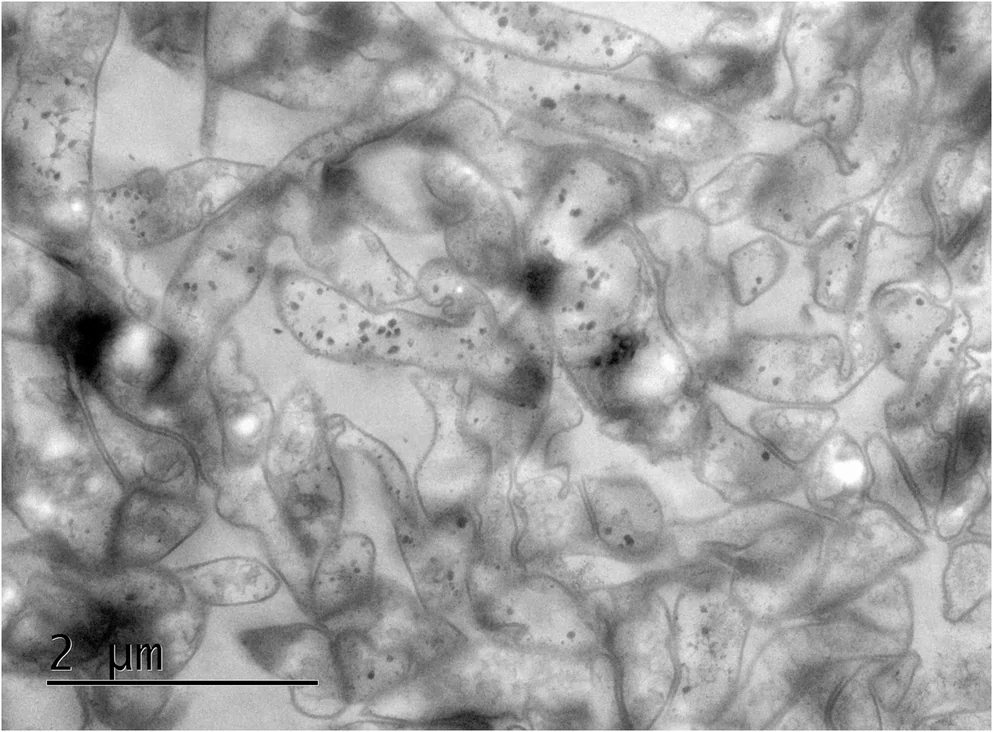
Transmission Electron Microscopy (TEM) image of 48 h sample of *S. eurocidicus* growth on glucose+soybean oil+casamino acids

On the TEM images (Fig. 4) there were no oil filled vesicles identified but electro-dense spots showed up dispersed in the cytoplasm and outside of the membrane.

**Fig. 5:**
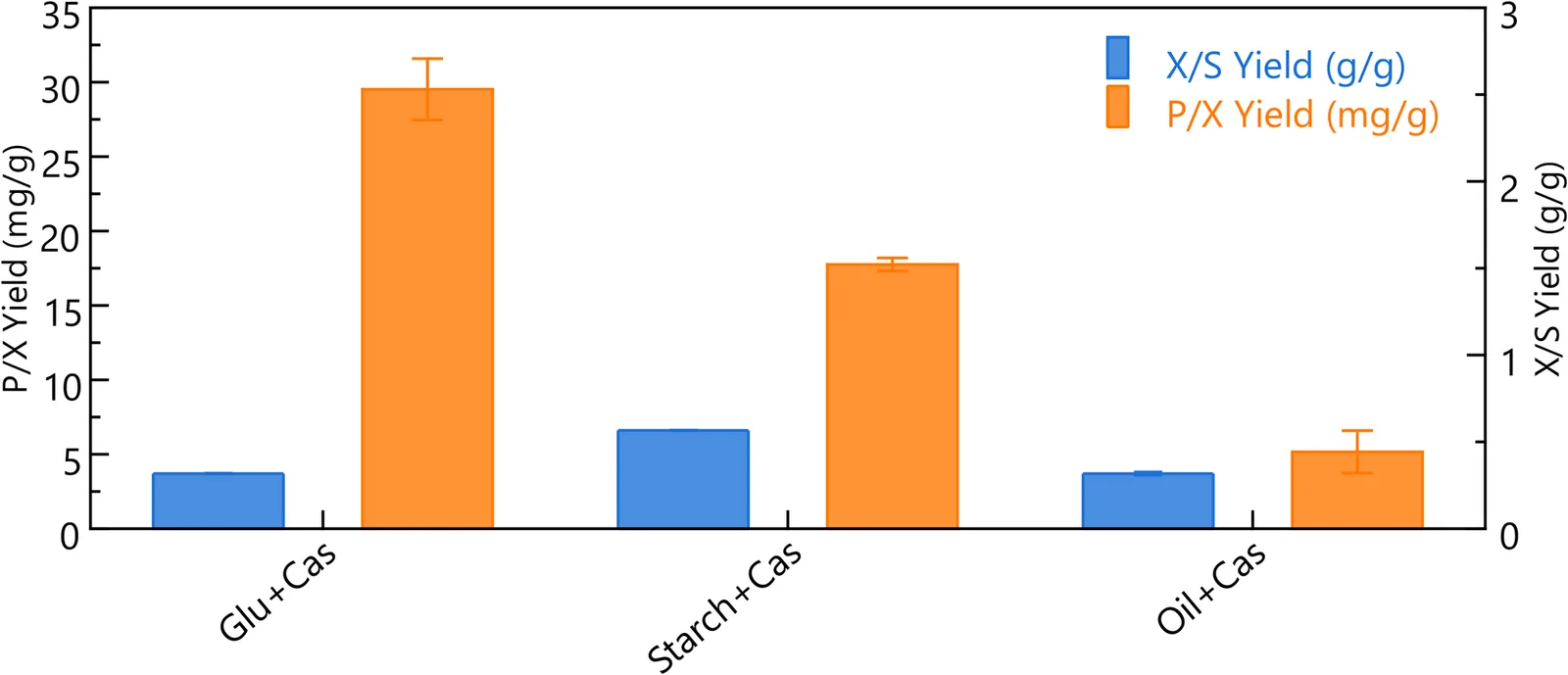
Azomycin per biomass (orange) and biomass per substrate (blue) yields obtained after 168 h of growth on minibiorector using different carbon sources: (1) Glucose 5 g/L and casamino acids 5 g/L; (2) Starch 5 g/L and casamino acids 5 g/L; (3) Soybean oil 5 g/L and casamino acids 5 g/L. Exact numerical values are provided in Table S2 for comparison

Carbon sources were also tested individually in the presence of casamino acids, and the corresponding yields are presented in Fig. 5.

**Fig. 6:**
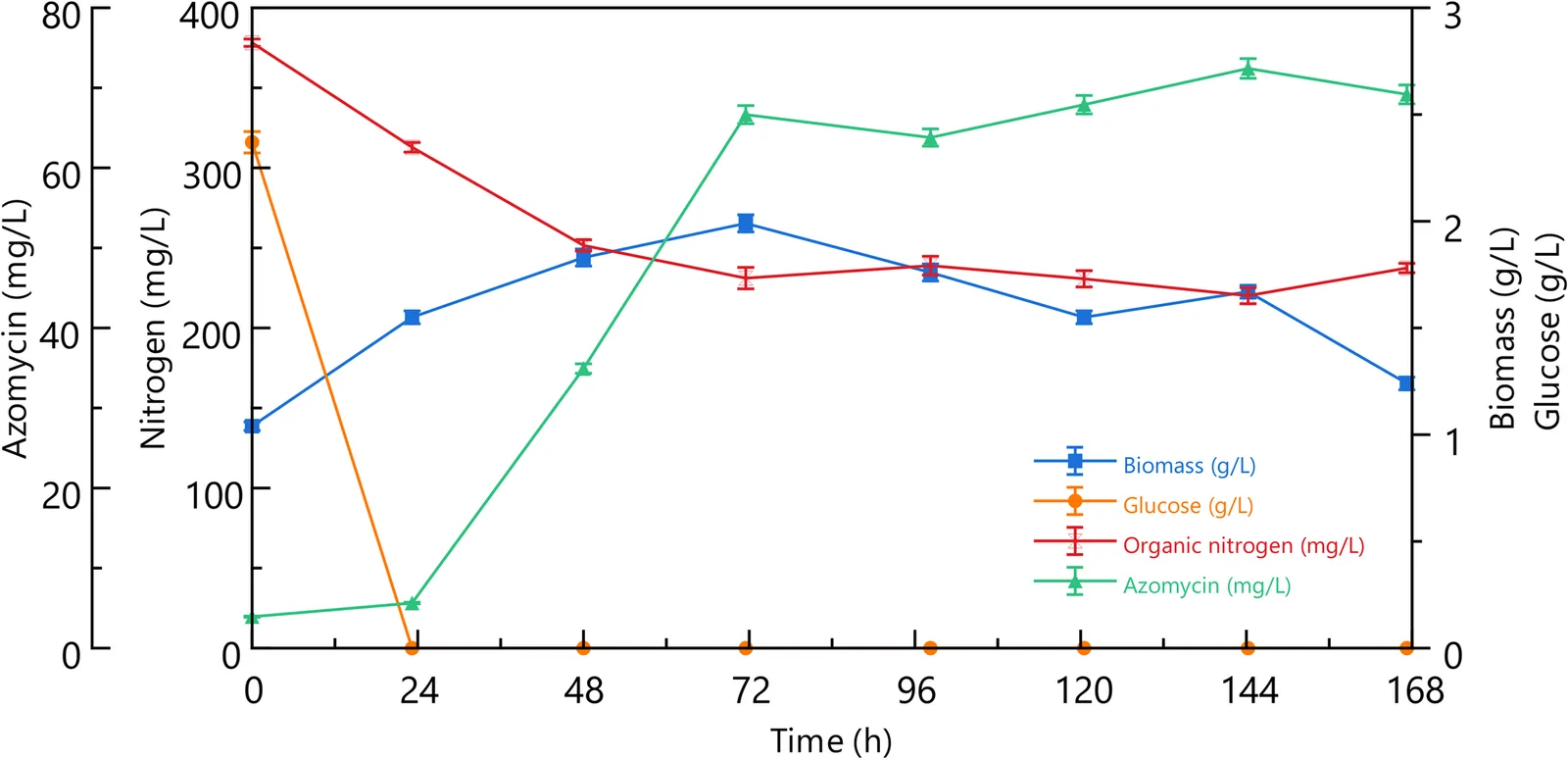
Cell growth, glucose and nitrogen consumption and azomycin production through 168 h of *S. eurocidicus* growth on 3 L bioreactor with semi-defined media using 2.5 g/L glucose as carbon source. Data from a single run, error bars calculated by triplicate analysis. Concentrations shown as: ■ Biomass (g/L); ● Glucose (g/L); ⧖ Nitrogen (mg/L);▲ Azomycin (mg/L)

When evaluating the azomycin per biomass yield, these results were significantly higher than the previously studied conditions, even though soybean oil remains returning the lowest results regarding production (5.16 mg/g). While the yield biomass per substrate is equivalent, a 5.7-fold decrease occurs by exchanging glucose for soybean in the product per biomass yield. In this comparison, starch provided a higher biomass (3.8 g/L) and titer (58.15 mg/L), but its Yp/x was 1.6-fold lower than from glucose. A comparison table between all conditions tested, including Yp/s is available on Table S2. The final titer from the semi-defined media with glucose and casamino acids was 46.34 mg/L and this condition was used to a follow-up cultivation in a 3 L reactor with full kinetics. Growth curve, carbon source consumption, organic nitrogen consumption and azomycin production obtained up to seven days cultivation is shown in Fig. 6.

**Fig. 7:**
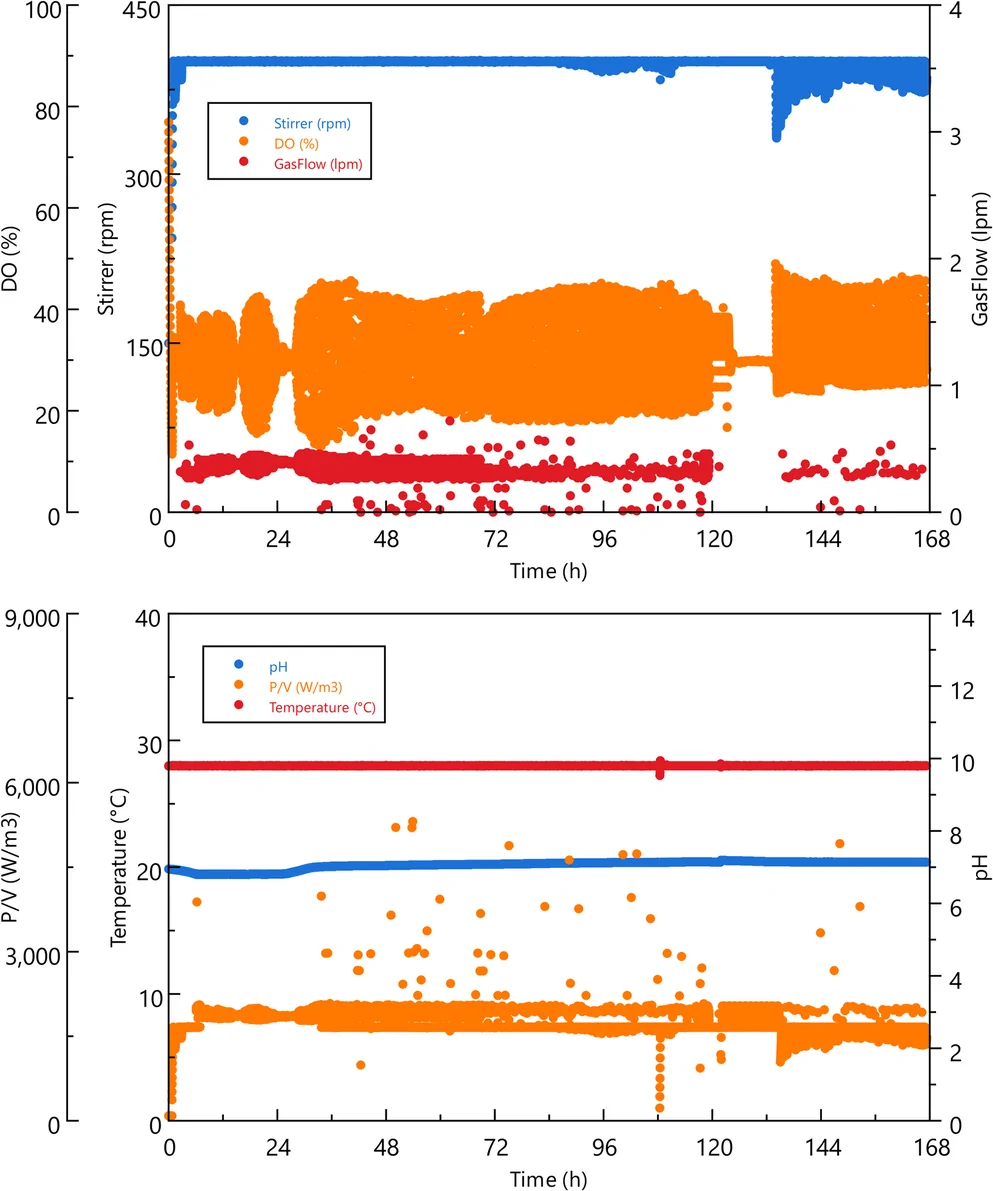
Trends of stirrer, gasflow, dissolved oxygen, temperature and pH obtained in 3 L bioreactor during the cultivation of *S.eurocidicus* in semi-defined media with 2.5 g/L glucose as carbon source. P/V was calculated

The complete batch proceeded with 2.5 g/L glucose instead of 5 g/L since previous experiments in minibioreators indicated that lower glucose had a higher outcome in regard to azomycin production (yields shown on Table S2 and stirring, aeration and DO are shown in Fig S3). Figure 7 shows stirring speed, gasflow, DO, pH, temperature and calculated P/V across this 168 h cultivation.

The stirrer reached the maximum speed allowed by the control system within the first 3 h. After that, DO was successfully controlled, oscillating between 20% and 40%, through increments in gasflow. Nevertheless, the maximum air flow set point was not reached during the cultivation time course, remaining around 0.5 lpm (0.17 vvm). Temperature and pH were tightly controlled. There was a slight temperature oscillation that lasted 15 min due to infrastructural issues; however, as it occurred around 108 h, when azomycin production had already reached its peak, there was no interference with the calculated yields.

The biomass per substrate yield on the 3 L bioreactor kept the same level obtained on the mini-bioreactor (around 0.4 g/g) with a specific growth rate of 0.01 h^− 1^. Azomycin production exceeded expectations after this scale up. On day seven, the total concentration achieved was 69.18 mg/L which meant a Yp/x of 55.90 mg/g, approximately 89% increase relative to the minibioreactor.

**Fig. 8:**
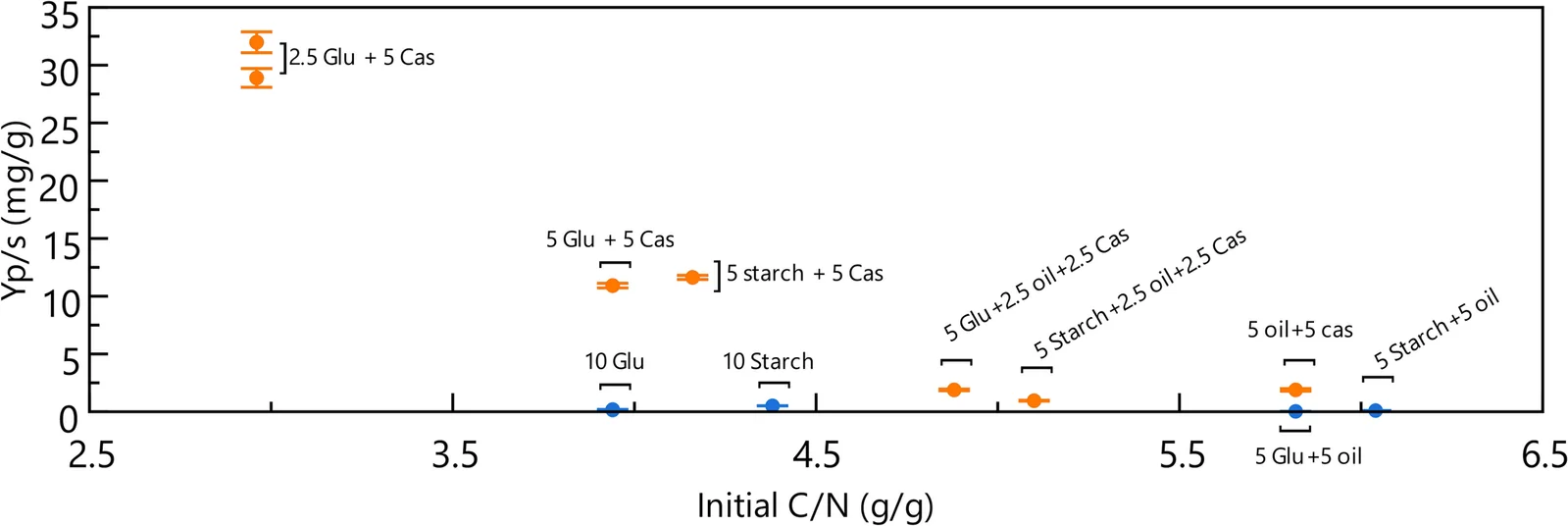
Calculated C/N ratio *versus* azomycin per substrate yield across all media combinations studied. Orange points highlight the conditions where casamino acids are present

Figure 8 shows the correlation between azomycin per substrate yield and the calculated initial C/N ratio.

All media without casamino acids presented low Y_P/S_ regardless of the initial C/N ratio. In contrast, among the media containing casamino acids there is a tendency for lower C/N ratios to result in higher yields. The presence of oil appears to be detrimental to azomycin production, as, when only oil-containing media are considered, a lower C/N ratio did not translate into increased yield.

**Fig. 9:**
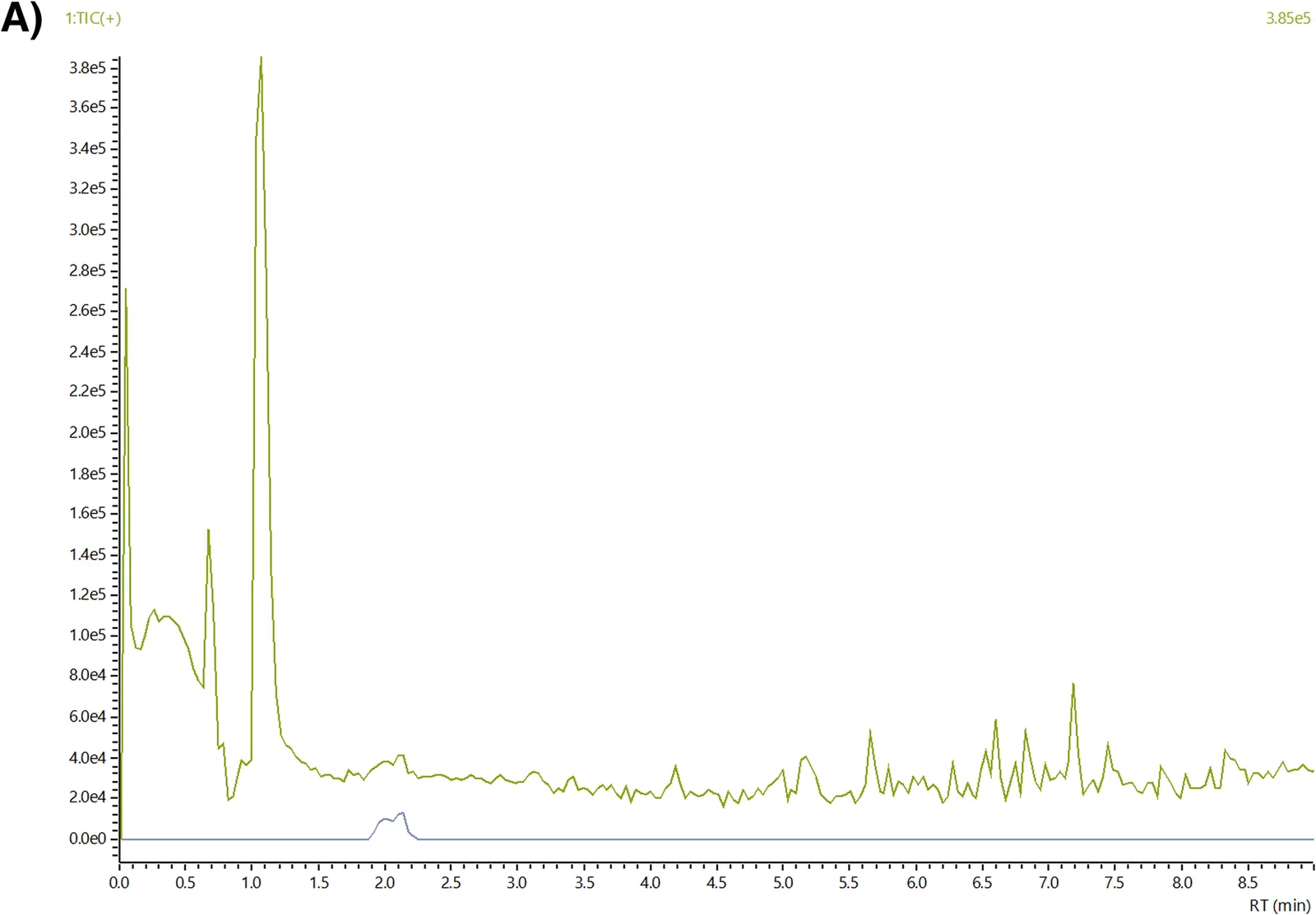

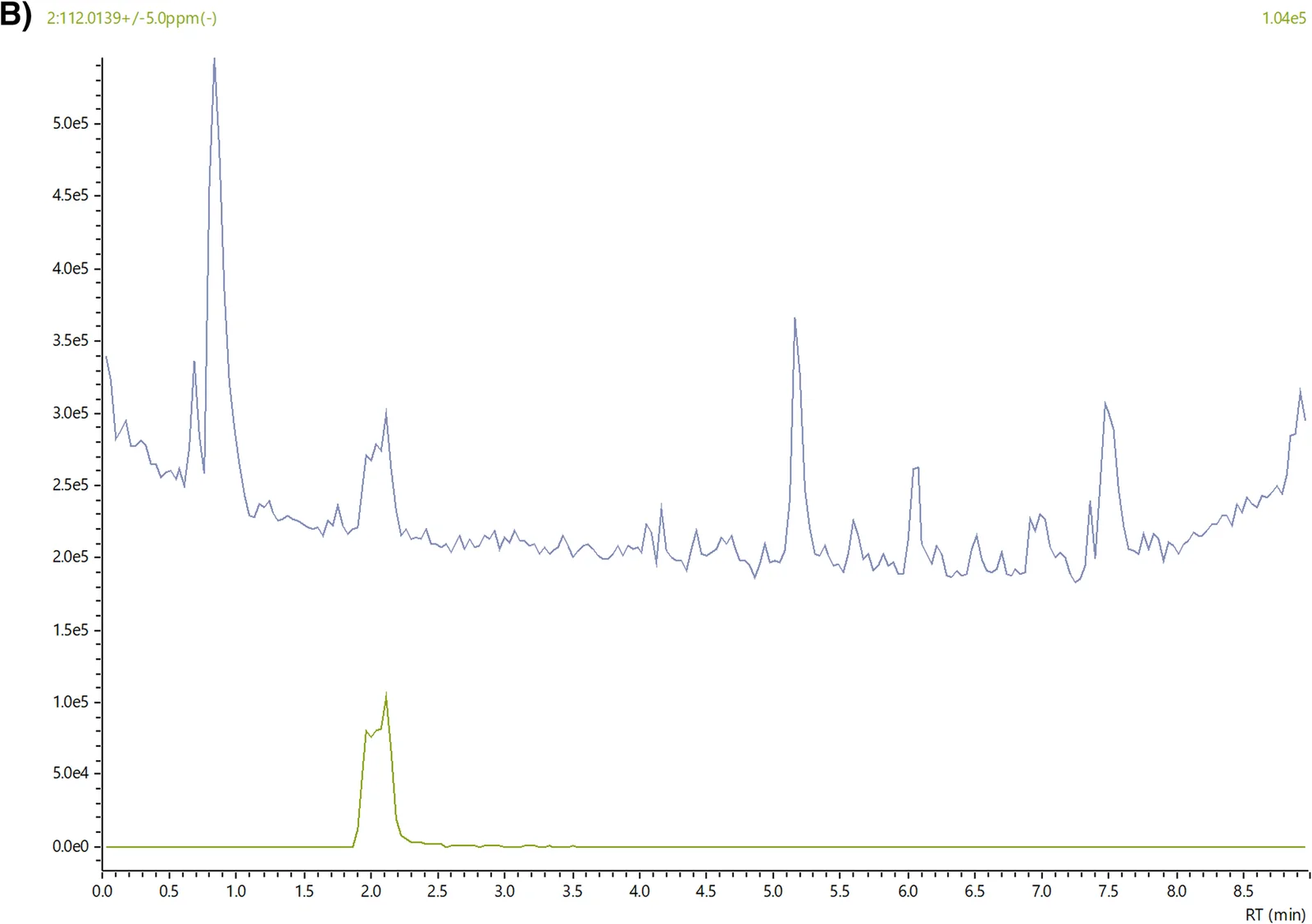
Extract analyses in positive mode (**a**) and negative mode (**b**). TIC is the first line, EIC is the second line

To identify and confirm the metabolite produced, extracts from the fermented broth underwent LC-MS analysis in both positive and negative ionization modes (Fig. 9). The total ion chromatograms (TIC) exhibited a higher number of peaks in positive mode compared to negative mode. However, extracted ion chromatograms (EIC) in both modes revealed mass values compatible with 2-nitroimidazole ionization: *m/z* 114.03 ([M + H]^+^) in positive mode and *m/z* 112.01 ([M-H]^−^) in negative mode.

**Fig. 10:**
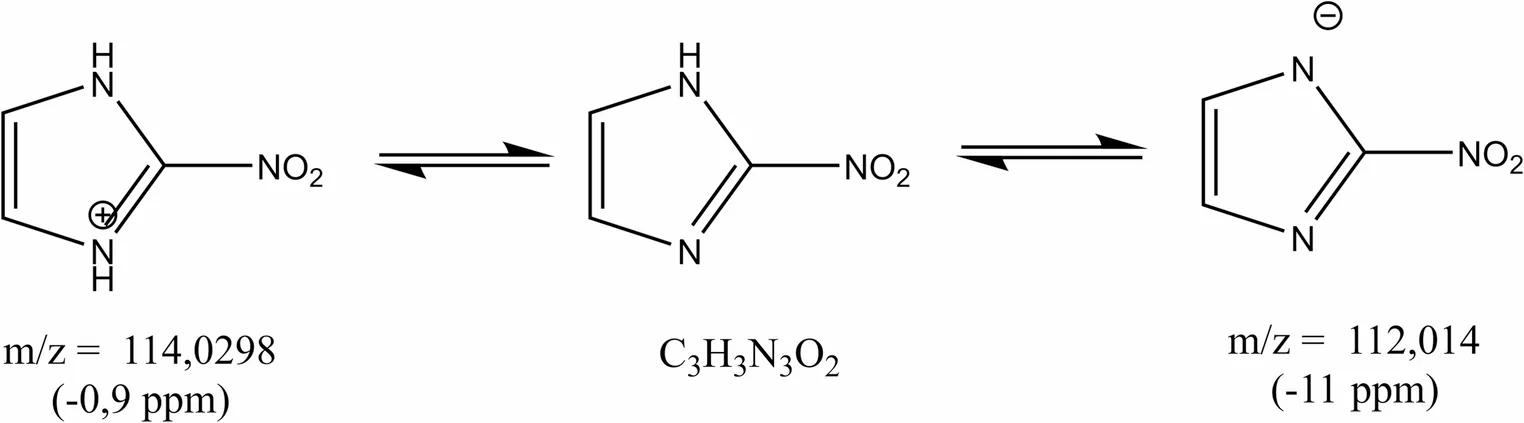
2-nitroimidazole (center) ionization forms. In the left protonated form; In right deprotonated form. Measured mass and associated error are also shown

The retention time (Rt) of 2-nitroimidazole was approximately 2 min, due to its high polarity and low affinity for the stationary phase. While 2-NI ionized in both conditions (Fig. 10), the signal intensity was stronger in negative mode (Fig. 9b), indicating that deprotonation is preferred over protonation for this molecule. Although this difference affected the mass error (which was lower in positive mode) both modes confirmed the expected molecular formula (C_3_H_3_N_3_O_2_).

## Discussion

The limited studies on the production of classical metabolites such as azomycin by *S. eurocidicus* makes it a compelling subject of investigation, especially since this species is already explored for other biological activities, including algicidal action, plant growth promotion and biocontrol for crops [24–26]. As the production of azomycin was reported in the literature [13], it is important to understand how the strain performs in liquid cultures and how carbon source selection affects yield.

Antibiotic production in *Streptomyces* is phase-shifted and regulatory-coupled from the primary metabolism as it depends on precursors and energy from it. As seen in Fig. 1, this behavior is also observed for *S. eurocidicus*. Alam and collaborators [27] studied the metabolic switch in *Streptomyces coelicolor* and demonstrated that the gene cluster responsible for antibiotics production is highly activated after the depletion of phosphates in the media which happens at a similar time than glucose depletion.

Recently the biosynthetic pathway for azomycin was elucidated by Hedges and Ryan [15], establishing that L-arginine is precursor of azomycin and identifying the enzymes related to this pathway. Even though amino acids are synthesized as a fundamental part of *Streptomyces* metabolism, supplying them in the medium can increase precursor availability and influence nitrogen balance and global regulator networks that control secondary metabolism [28–31]. The other modification in the media, involving addition of soybean oil, took into consideration that the lipid metabolism of *S. eurocidicus* has not been characterized. However, Peacock and collaborators demonstrated that in *Streptomyces lividans* the use of triolein and glucose as mixed substrates increased the productivity, allowing the culture to reach the maximum of actinorhodin production by the fifth day instead of the eighth, although the maximum concentration was similar in both cases [32]. Other *Streptomyces* species also consume fatty acids and long-chain n-alkanes as sole carbon and energy sources [33–35], including the formation of visible internal bodies when the fatty acids are intracellular [35]. These internalized lipids, mostly in form of triacylglycerols (TAG) and other neutral lipids, are stored surrounded by a phospholipid monolayer that can occupy up to 80% of cell volume and are used as energy reserves for when external nutrients are low [36–38]. These reserves could be used to extend the production phase of the target secondary metabolite which could lead to increased production yields of azomycin. Another advantage of having an oil in the media composition is its ability to act as a physical antifoam which is undesirable for secondary metabolites production since it makes gas exchange difficult, impacting negatively on both growth and production [39, 40]. Nevertheless, in this study soybean oil was found to be a good carbon source for biomass production, but not a viable option for azomycin production as there was no production. This result contrasts with findings for *Streptomyces albus* by Li and collaborators [41] where salinomycin production was nearly basal when growing solely on glucose while production increased almost four-fold when using soybean oil. That study also observed increased biomass production comparable with present results.

Mini-bioreactors were used to screen different carbon sources and their combinations, allowing a larger number of conditions to be tested. Because of their low working volume, proper kinetic profiling was not possible: the azomycin extraction and analysis methodology used in this study required at least 30 mL per sample, so only initial and final samples were taken from the mini-bioreactors. The results from this screening were used to select the medium composition with the best azomycin production outcome, which was then carried forward to the 3 L scale chosen for kinetics study. The 3 L working volume was selected because it allowed repeated 30 mL sampling over several days without a significant reduction in culture volume. Comparisons between the 250 mL and 3 L results are not intended as a strict scale-up: stirring and aeration were not adjusted between scales, as the cascade control used to maintain the dissolved oxygen controlled at the setpoint (30%) adjusts stirrer and adds compressed air as requested by the microorganism.

On Fig. 2 comparison among glucose-based media shows the presence of oil as detrimental for azomycin production but when casamino acid was added, production increased three-fold. In the media with starch only, adding oil was also detrimental, and the presence of casamino acid was insufficient to overcome this effect, as the Yp/x obtained was 26% lower than starch alone. This result reinforces the benefits of having free amino acids in the culture for antibiotics production as reported for other *Streptomyces* species [28, 31]. As casamino acids is supplying the direct biosynthetic precursor of 2-nitroimidazole, their inclusion in the medium appears to increase the intracellular pool of L-arginine, thereby supporting a higher flux through the azomycin pathway rather than relying on intrinsic arginine biosynthesis from inorganic nitrogen sources such as ammonium sulfate, as used in the conditions where casamino acids were not present.

The additional characteristic that emerged on these cultivations is that crystals could be observed in cultivations that contained casamino acids. They exhibited a rectangular morphology resembling the azomycin reference under the SEM conditions employed. However, morphology alone is insufficient for chemical identification. Considering the measured azomycin concentration, the uncertain solubility of 2-nitroimidazole in fermentation broth, the possibility of local supersaturation adjacent to the producing mycelium that may influence, and the presence of phosphate and divalent metal ions the particles may comprise azomycin, inorganic salts, or mixed precipitates. Definitive assignment will require direct characterization of isolated particles by techniques such as SEM-EDS, micro-Raman or FTIR spectroscopy and powder X-ray diffraction. Also, there is appearance of electro-dense spots on TEM image (Fig. 4) that matches the start of azomycin production and should be further investigated to confirm if they are correlated to crystals observed in SEM images from Fig. 3a. Also, it was not possible to identify oil filled vesicles as was expected based on Packter and Olukoshi [37] results on *S. lividans*.

A final comparison testing individually each carbon source (glucose, starch and soybean oil) in the semi-defined media with casamino was performed and the highest final titer (46.34 mg/L) was obtained with semi-defined with glucose and this was the condition chosen to evaluate a batch process in a 3L bioreactor within 7 days process (Fig. 6). It’s important to notice that, even though the two volumes were not designed as a scale up because of the sample volume constraint, the P/V obtained in both volumes is different and may influence the final azomycin titer obtained. For example, at the production peak, P/V at 250 mL was 503 W/m^3^ while at 3L was 2,007 W/m^3^, which is four-fold higher (Fig S3 and Fig. 7). Since specific power input (P/V) was not matched between scales, the higher P/V may have enhanced mixing and oxygen transfer, and this should be evaluated in further dedicated experiments in which this parameter is controlled across scales. The value of specific growth rate found on the first comparison batch of this study (Fig. 1) was 0.03 h^− 1^ which matches the expected values for *Streptomyces lividans* on a liquid minimal medium with glucose as the sole carbon source [42]. Nevertheless, the growth rate on glucose+casamino acids was 0.01 h^− 1^ (Fig. 6), lower than expected as growth on mixed substrates has not been reported as a reason for lower growth rate, instead rather the opposite [32].

When comparing C/N ratios of media containing glucose and starch, no significant difference was observed. However, across all media compositions tested, a tendency of increased azomycin per substrate yield was observed at lower C/N ratios. This behavior contrasts with the commonly reported model in which nitrogen limitation (high C/N ratio) triggers antibiotic production because it halts growth and redirects flux to secondary pathways [43, 44]. Nevertheless, a recent study done by Meng and collaborators (2025) [45] found that cultivation in a medium rich in organic nitrogen increased natamycin titer by 2.8-fold when compared with same conditions using inorganic nitrogen. This difference was attributed to the upregulation of NAD(P) biosynthetic genes and increased NAD/NADP pools. In the case of azomycin production investigated in this study, there is also the additional benefit that its biosynthesis originates from L-arginine. Thus, lowering the C/N through the addition of casamino acids provides a carbon backbone for the imidazole ring, reducing the need for the cells to biosynthesize *de novo* arginine from ammonium and α-ketoglutarate [15, 46]. The exact mechanism underlying the increase in Y_P/S_ and the lower growth rate observed under these conditions remains to be elucidated.

## Conclusion

This study characterized the physiological and nutritional requirements for azomycin (2-nitroimidazole) production by *S. eurocidicus* under controlled submerged cultivation. The findings demonstrate that azomycin biosynthesis is distinctly phase-shifted, initiating only after near depletion of primary carbon source and slowing exponential growth.

Furthermore, media composition critically dictates the metabolic fate of the strain. While the use of soybean oil as a carbon source effectively doubled biomass accumulation, it completely repressed the production of azomycin. Conversely, supplementation with organic nitrogen in the form of free amino acids (casamino acids) proved essential for stimulating secondary metabolism, overcoming inhibitions, and substantially enhancing antibiotic yields.

Ultimately, scale-up in a 3 L bioreactor using an optimized semi-defined medium of glucose and casamino acids successfully yielded a maximum titer of 69.18 mg/L on day seven, representing an 89% increase in product-to-biomass yield (Y_p/x_) compared to the minibioreactor scale. Also, results showed that lower C/N ratio, with the enrichment of an organic nitrogen source, outcomes higher azomycin titers. These results establish that precise balancing of carbon and nitrogen sources is critical to unlock the biosynthetic potential of wild-type *S.eurocidicus*, and provide an effective, scalable strategy for industrial azomycin production.

This study has limitations arising from a design intended to compare carbon and nitrogen source composition. The contribution of differences in P/V to the higher azomycin production observed at the 3 L scale, potentially through enhanced mixing and/or oxygen mass transfer, cannot be excluded based on the present data. Further experiments with power matched across scales are therefore needed to quantify this effect.

## Supplementary Information

Below is the link to the electronic supplementary material.

Supplementary Material 1

## Data Availability

The datasets generated and analyzed during the current study are available from the corresponding author on reasonable request.
