# Supplementary material for: Novel insights on carbon source influence on azomycin production by Streptomyces eurocidicus
Source: Braz J Microbiol. 2026 Oct 12;57(1):290. doi: 10.1007/s42770-026-02102-8 (PMC13658643; doi:10.1007/s42770-026-02102-8)
Supplement: Supplementary file 1 [file 42770_2026_2102_MOESM1_ESM.pdf]

Supplementary Information

Novel insights on carbon source influence on azomycin production by *Streptomyces eurocidicus.*

Brazilian journal of microbiology

Julia de Macedo Robert [1*], Wagner Lopes[1], Alexandre Andrade de Souza Costa[1], Otavio Padula de Miranda[1], Tatiane Aparecida Nascimento[2], Maria da Conceição Avelino Dias[3], Filipe Soares Quirino da Silva[3], Fabius Vieira Leineweber[1], Graziela Jardim Pacheco[1].

[1] Bioprocess Laboratory, Institute of Drug Technology, Oswaldo Cruz Foundation, Fiocruz

[2] Integrated Laboratory of Simuliids and Onchocerciasis & Medical and Forensic Entomology, Oswaldo Cruz Institute, Oswaldo Cruz Foundation, Fiocruz

[3] Drug Synthesis Laboratory – Pharmacokinetics Unit, Institute of Drug Technology, Oswaldo Cruz Foundation, Fiocruz

[*]Corresponding author: julia.robert@fiocruz.br / ORCID: https://orcid.org/0000-0003-1945-4750

**Corresponding author: graziela.pacheco@fiocruz.br / ORCID: https://orcid.org/0000-0002-7493-7969

Supporting Information contains 6 Pages, 3 Figures, 2 Tables.

Relatório de Experimento- AZO-003/26 -13/03/26

Arquivo de Dados: C:\Enterprise\Projects\AZOMICINA\2026\03 Março\Result\2026.03.13_AZO.03 E AZO.04.rslt\PAD AZOM 0.22mg-Rep1.mL
Método: C:\Enterprise\Projects\AZOMICINA\2026\03 Março\Method\AZOMIC_YMC C18 5um 250x4.6_Meth.met
Adquirido: 03/03/2026 02:17:13 (GMT -03:00) AMOSTRA: PAD AZOM 0.22mg.mL VIAL= 13
Impresso: 03/03/2026 17:50:20 (GMT -03:00) Vol. Injeção = 5 uL

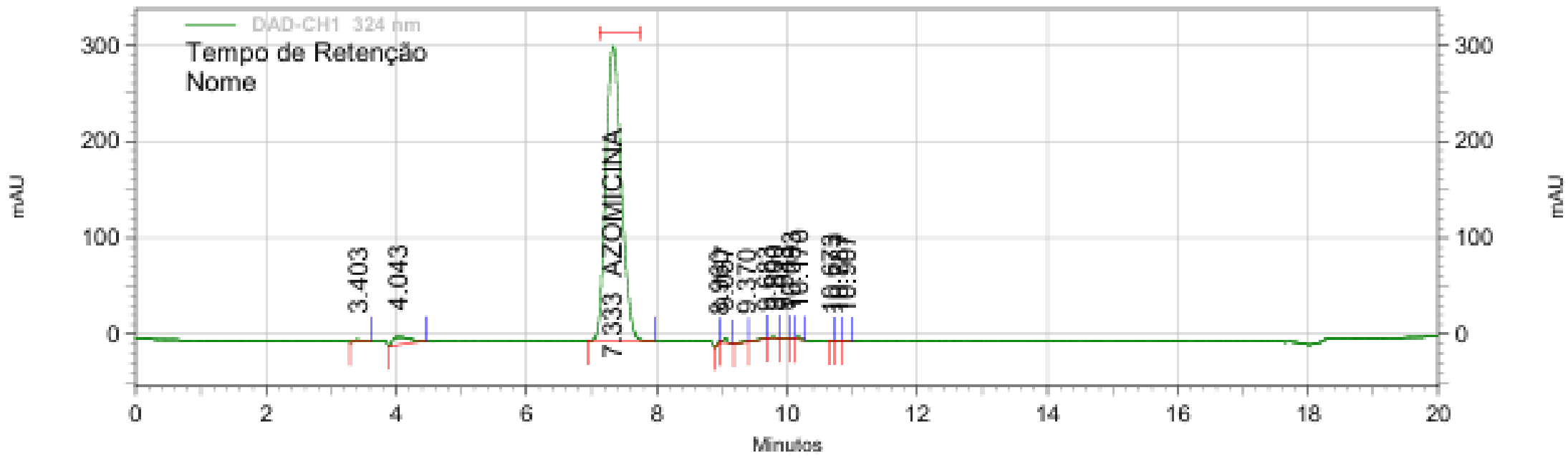

Resultados DAD-CH1 324 nm

Tempo de Retenção	Nome	Área	Percentual de Área	Largura	Pratos teóricos (USP)	Assimetria	Concentração ESTD
3.403		71276	0.341	0.33	2765	1.489	0.000
4.043		566875	2.713	0.56	1339	1.856	0.000
7.333	AZOMICINA	20012497	95.761	1.04	4582	1.142	0.000 Padrão
8.960		20504	0.098	0.07	302731	0.656	0.000
9.057		76274	0.365	0.18	165246	0.973	0.000
9.370		20472	0.098	0.23	155734	0.589	0.000
9.683		22060	0.106	0.29	18177	0.536	0.000
9.800		35256	0.169	0.18	66855	0.884	0.000
9.943		26629	0.127	0.15	90996	1.355	0.000
10.083		5020	0.024	0.08	473045	0.896	0.000
10.170		36078	0.173	0.14	290240	1.297	0.000
10.673		769	0.004	0.09	333580	1.188	0.000
10.787		2263	0.011	0.13	172859	1.001	0.000
10.907		2451	0.012	0.14	141974	1.228	0.000
Totais		20898424	100.000				0.000

Fig S1 Report from HPLC for 95% pure 2-nitroimidazole standard reference.

Relatório de Experimento- AZO-004/26 -18/03/26

Arquivo de Dados: C:\Enterprise\Projects\AZOMICINA\2026\03 Março\Result\2026.03.18 AZO 04.CONT.rslt\P7 AZO 4.dat
Método: C:\Enterprise\Projects\AZOMICINA\2026\03 Março\Method\AZOMIC_YMC C18 5um 250x4.6_Meth.met
Adquirido: 06/03/2026 02:44:14 (GMT -03:00) AMOSTRA: P7 AZO 4 VIAL= 25
Impresso: 06/03/2026 05:02:28 (GMT -03:00) Vol. Injeção = 5 uL

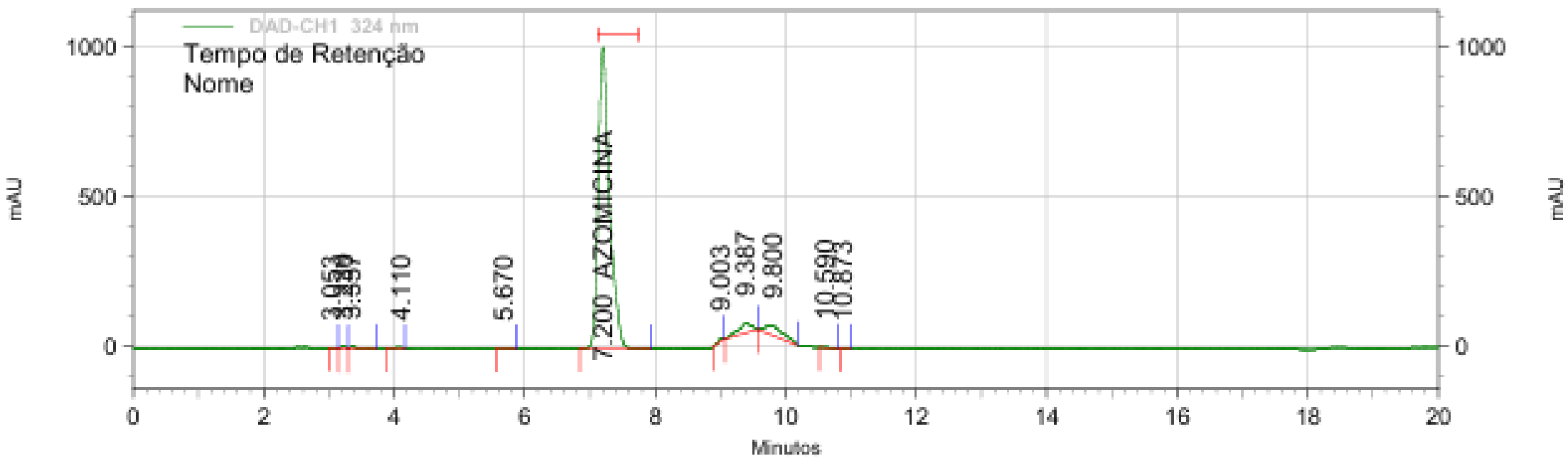

Resultados DAD-CH1 324 nm Tempo de Retenção	Nome	Área	Percentual de Área	Largura	Pratos teóricos (USP)	Assimetria	Concentração ESTD
3.053		30421	0.057	0.13	11396	1.481	0.000
3.230		163838	0.308	0.15	9481	0.859	0.000
3.357		222299	0.418	0.44	6643	3.096	0.000
4.110		142277	0.267	0.29	4601	0.615	0.000
5.670		7965	0.015	0.30	10358	1.528	0.000
7.200	AZOMICINA	48045396	90.244	1.10	8869	1.396	0.000 Padrão
9.003		202354	0.380	0.17	140119	0.695	0.000
9.387		1822552	3.423	0.51	11840	0.790	0.000
9.800		2564662	4.817	0.62	4934	1.381	0.000
10.590		35067	0.066	0.29	65712	1.997	0.000
10.873		2388	0.004	0.15	0	3.339	0.000
Totais		53239219	100.000				0.000

Fig S2 Report from HPLC for 168h sample of *S. eurocidicus* growth on 3L bioreactor with semi-defined media using 2.5 g/L glucose as carbon source.

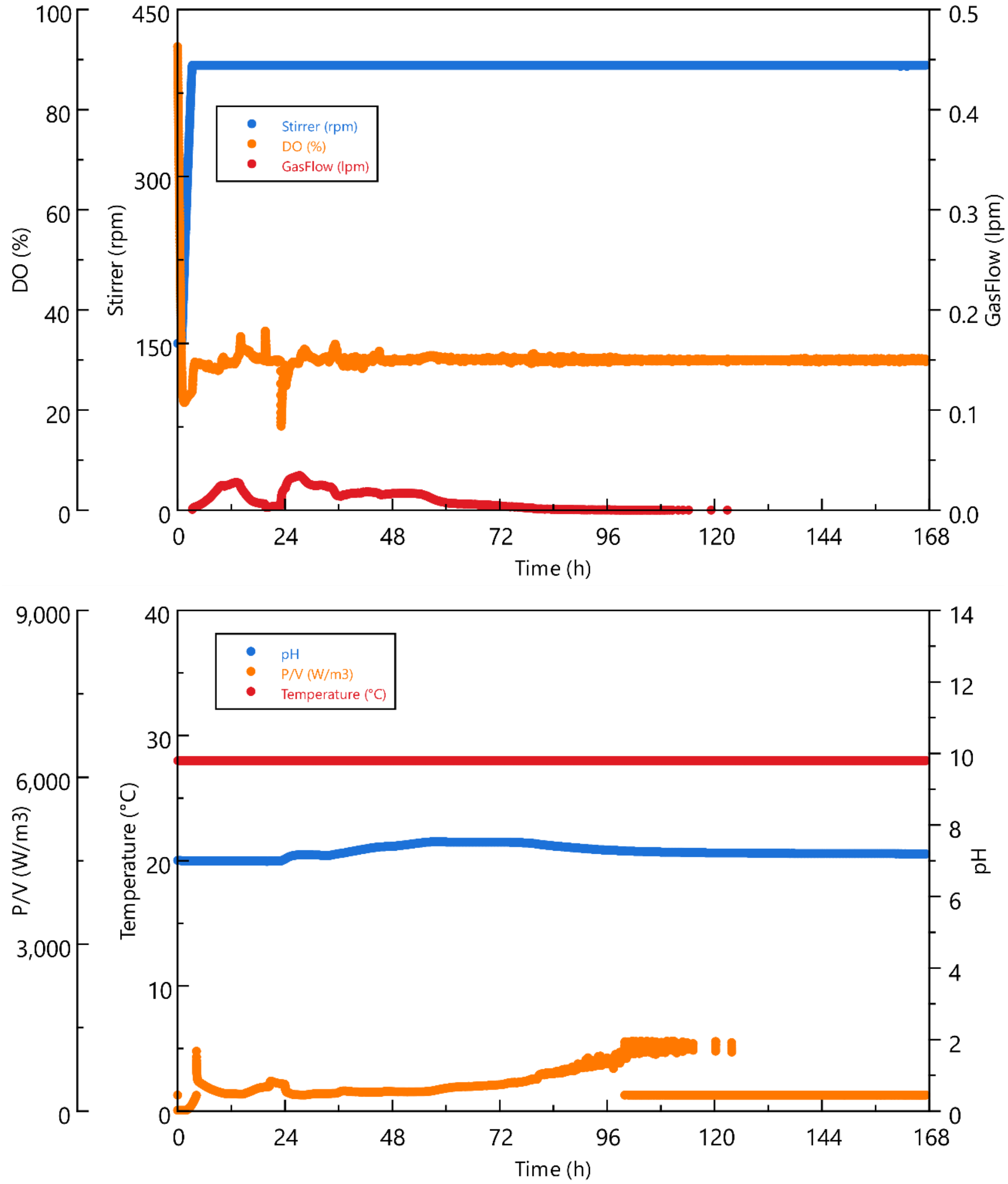

Fig S3 Trends of stirrer, gasflow, dissolved oxygen, temperature and pH obtained in 250 mL bioreactor during the cultivation of *S.eurocidicus* in semi-defined media with 2.5 g/L glucose+ 5 g/L casamino acids. P/V was calculated

Table S1 Comparison of azomycin production reports across *Streptomyces* strains in submerged fermentation.

Microorganism	Azomycin titer	Key finding	Reference
Streptomyces sp. LE/3342	± 100 µg/ml	Produced azomycin alongside eurocidin and tertiomycin	[1]
Streptomyces sp. (unspecified azomycin producer)	Increased yield but quantitative value not specified	Addition of 2-aminoimidazole boosted azomycin yields; >50% of radiolabeled precursor recovered in product	[2]
S. eurocidicus mf294	Not quantified	2-Nitroimidazole identified as a carbonic anhydrase inhibitor; complete inhibition at 1×10^{-5} M	[3]

1. Lancini CG, Lazzari E, Sartori C (1968) MICROBIAL OXIDATION OF AMINOIMIDAZOLES TO NITROIMIDAZOLES. J Antibiot (Tokyo) XXI:387–392. https://doi.org/https://doi.org/10.7164/antibiotics.21.387

2. Lancini GC, Kluepfel D, Lazzari E, Sartori G (1966) ORIGIN OF THE NITRO GROUP OF AZOMYCIN. Biochimica etBiophysica Acta 130:37–44. https://doi.org/https://doi.org/10.1016/0304-4165(66)90006-7

3. Fukushima M, Ozaki N, Ikeda H, Furihata K, Hayakawa Y, Sakuda S, Nagasawa H (2002) Isolation and Characterization of 2-Nitroimidazole Produced by Streptomyces Species as an Inhibitor of Both Carbonic Anhydrase and Shell Formation in the Barnacle Balanus amphitrite. Marine Biotechnology 103–110. https://doi.org/10.1007/s1012602-0091-5

Table S2 Biomass per substrate, azomycin per substrate and azomycin per biomass yields obtained at all conditions tested in this study.

	Scale	Initial C/N (g/g)	$Y_{X/S}$ (g/g)	$Y_{P/S}$ (mg/g)	$Y_{P/X}$ (mg/g)
**10 g/L Glucose**	3L	3.94	0.10 ± 0.0092	0.15 ± 0.0073	0.38 ± 0.0330
**10 g/L Glucose**	250 mL	3.94	0.17 ± 0.0358	0.19 ± 0.0027	1.14 ± 0.2478
**10 g/L Starch**	250mL	4.38	0.15 ± 0.0092	0.51 ± 0.0061	3.3 ± 0.2006
**5 g/L Glucose + 5 g/L Soybean oil**	250mL	5.82	0.17 ± 0.0049	0.03 ± 0.0005	0.08 ± 0.0022
**5 g/L Starch + 5 g/L Soybean oil**	250mL	6.04	0.20 ± 0.0133	0.1 ± 0.0012	0.26 ± 0.0173
**5 g/L Glucose + 2.5 g/L Soybean oil + 2.5 g/L Casamino acids**	250mL	4.88	0.26 ± 0.0219	1.89 ± 0.0697	3.36 ± 0.2596
**5 g/L Starch + 2.5 g/L Soybean oil + 2.5 g/L Casamino acids**	250mL	5.10	0.39 ± 0.0276	0.95 ± 0.0351	2.45 ± 0.1548
**5 g/L Glucose + 5 g/L Casamino acids**	250mL	3.94	0.37 ± 0.0260	10.92 ± 0.2042	29.52 ± 2.0643
**5 g/L starch + 5 g/L Casamino acids**	250mL	4.16	0.66 ± 0.0156	11.63 ± 0.1817	17.75 ± 0.4393
**5 g/L Soybean oil + 5 g/L Casamino acids**	250 mL	5.82	0.37 ± 0.1037	1.89 ± 0.1097	5.16 ± 1.4299
**2.5 g/L Glucose + 5 g/L Casamino acids**	3L	2.96	0.40 ± 0.0122	28.90 ± 0.8102	55.90 ± 1.4967
**2.5 g/L Glucose + 5 g/L Casamino acids**	250mL	2.96	0.79 ± 0.0313	31.98 ± 0.9030	40.56 ± 1.3219
